# Scrub typhus associated hepatic dysfunction and abdominal CT findings

**DOI:** 10.12669/pjms.312.6386

**Published:** 2015

**Authors:** Man Je Park, Hyoun Soo Lee, Sang Goon Shim, So Hee Kim

**Affiliations:** 1 Man Je Park, Department of Internal Medicine, Samsung Changwon Hospital, Sungkyunkwan University School of Medicine, Gyeongsangnam-do, Korea; 2 Hyoun Soo Lee, Department of Internal Medicine, Samsung Changwon Hospital, Sungkyunkwan University School of Medicine, Gyeongsangnam-do, Korea; 3 Sang Goon Shim, Department of Internal Medicine, Samsung Changwon Hospital, Sungkyunkwan University School of Medicine, Gyeongsangnam-do, Korea; 4 So Hee Kim, Department of Radiology, Samsung Changwon Hospital, Sungkyunkwan University School of Medicine, Gyeongsangnam-do, Korea

**Keywords:** Scrub typhus, *Orientia tsutsugamushi*, Hepatitis, Computed tomography

## Abstract

**Objective:**

This retrospective study investigated abnormal hepatic dysfunction and abdominal computed tomography (CT) findings in scrub typhus.

**Methods:**

Three hundred forty nine adult patients were diagnosed with scrub typhus. Ninety four underwent abdominal CT. The CT images were reviewed by the attending radiologist. Patient data of history, symptoms, signs, and results of laboratory tests were collected from the electronic medical records.

**Results:**

In 349 patients with scrub typhus, elevation of aspartate aminotransferase (78.5%) and alanine aminotransferase (63.0%) were dominant compared to alkaline phosphatase (27.2%) and total bilirubin (16.1%). Abdominal CT findings of 94 patients were, in descending order of frequency, enlarged lymph node (53.2%), inhomogeneous enhancement of liver (47.9%), splenomegaly (46.8%), ascites (28.7%), low attenuation of periportal areas (27.7%), gallbladder wall thickening (17.0%), and splenic infarct (6.4%). Also, the level of aspartate aminotransferase tended to be elevated according to the number of CT findings (*P*= 0.028)

**Conclusions:**

We found that abdominal CT manifestations of scrub typhus with elevated aminotransferases were varied and not specific. However, knowledge of these findings may evoke the recognition of scrub typhus by clinicians in endemic areas.

## INTRODUCTION

Scrub typhus is a mite-borne infectious disease caused by *Orientia tsutsugamushi*. The disease is one of the acute febrile illnesses in Korea during autumn and is manifests with high fever, headache, myalgia, and, in many patients, rash and an eschar. It usually involves multiorgan including liver.^1^,^2^ It is known that the hepatic dysfunction in patients with scrub typhus is quite common (70-90%).^3^-^6^ The diagnosis of scrub typhus is usually based on the history of exposure, clinical features, and serologic assay. However, in some cases, the diagnosis can be complicated when there is difficulty in finding an eschar or in the absence of serologic test.^7^,^8^

When febrile patients with hepatic dysfunction visit an emergency room or outpatient clinic, abdominal computed tomography (CT) may be considered for differential diagnosis. Few studies described abdominal CT features of scrub typhus with hepatic dysfunction to date. Jeong et al. in 2007 first demonstrated the abdominal CT findings of scrub typhus; splenomegaly, periportal areas of low attenuation, inhomogeneous enhancement of liver, gallbladder wall thickening, lymphadenopathy, splenic infarct, and ascites.^9^ However, the number of cases was small and it was uncertain whether CT findings were assedded with particular reference to hepatic dysfunction. Thus, we aimed to investigate abdominal CT findings in patients who had scrub typhus with abnormal aminotransferase activity.

## METHODS

### Patients

This retrospective study was conducted between July 2008 and December 2013 at Samsung Changwon Hospital, Changwon, Korea. During this 5-year and 6-month period, 359 patients over 19 years of age were diagnosed with scrub typhus. The diagnosis was made if any one of the following criteria were met: (1) history of outdoor exposure, (2) pathognomonic eschar, and (3) single measurement of serologic test at the initial visit.^7^,^8^ The serologic test for scrub typhus was conducted using a commercial immunochromatographic test (ICT) (Tsutsugamushi assay; SD Bioline, Youngin, Korea), with a positive or negative result.^10^ The exclusion criterion was a history of previously known liver disease (e.g. chronic viral hepatitis, liver cirrhosis, or malignancy of liver). Ten of 359 patients diagnosed with scrub typhus were excluded due to liver cirrhosis (n=4), chronic hepatitis B (n=4), chronic hepatitis C (n=1), and Klatskin tumor (n=1). The symptoms, signs, and results of the initial laboratory tests of 349 patients were investigated through examination of the electronic medical records.

### Abdominal CT

Abdominal CT was taken within one week after the manifestation of the symptoms or signs of abdominal distress, especially abdominal pain, or hepatic dysfunction with fever. 94 of the 349 patients underwent abdominal CT. One radiologist retrospectively reviewed the CT images. We referred to the literature about abdominal CT findings of scrub typhus and concentrated on the following findings: (1) enlarged lymph node, (2) inhomogeneous enhancement of liver, (3) splenomegaly, (4) ascites, (5) low attenuation of periportal areas, (6) gallbladder wall thickening, and (7) splenic infarct.^9^ Enlarged lymph node was defined as abdominopelvic lymph node with short-axis diameter of more than 10 mm. Splenomegaly was defined as a maximal width of more than 11 cm on axial CT scan.^11^

### Ethical considerations

This study was reviewed and approved by the institutional ethical review committee of Samsung Changwon Hospital, Sungkyunkwan University.

### Statistical analyses

All values were expressed as the mean ± standard deviation (SD) or median with range. Comparison of baseline parameters according to the number of CT findings was evaluated statistically by the one-way ANOVA and the Kruskal-Wallis test after the Kolmogorov-Smirnov test for normality. Aspartate aminotransferase (AST) and alanine aminotransferase (ALT) were log transformed for parametric statistics. Post-hoc analysis was done by the Bonferroni correction. *P*< 0.05 was considered statistically significant. Analyses were performed using SPSS version 19.0 for Windows (SPSS Inc., Chicago, IL, USA).

## RESULTS

### Baseline characteristics

Male was 145 (41.5%), Female 204(58.5%). The mean age was 63.7 ± 16.2 years. Clinical manifestations were fever (92.8%), chills (78.5%), rash (59.0%), myalgia (57.3%), headache (40.1%), nausea or vomiting (19.5%), cough (18.9%), abdominal pain (18.6%), shock (4.3%), impaired mental health (3.2%), and lymphadenitis (1.7%). An eschar was detected in 245 of 349 patients (70.2%). ICT was positive in 265 of 349 patients (75.9%) (Table-I).

**Table-I:** Baseline characteristics of the 349 patients with scrub typhus.

Characteristic	Median (range)	Proportion of abnormality
Male	145 (41.5)	
Female	204 (58.5)	
Age	63.7 ± 16.2	
AST (IU/L)	71 (12 –>2600)	274/349 (78.5)
ALT (IU/L)	55 (9 –>2600)	220/349 (63.0)
ALP (IU/L)	88 (17 – 1492)	94/346 (27.2)
Total bilirubin (mg/dL)	0.8 (0.2 –>30.0)	56/347 (16.1)
Albumin (g/dL)	3.2 (1.7 – 4.5)	120/346 (34.7)
PT(INR)	1.00 (0.26 – 8.05)	9/295 (3.1)
White blood cells (/μL)	6400 (1100 – 26900)	150/348 (43.1)
Hemoglobin (g/dL)	12.6 (8.5 – 17.9)	114/348 (32.8)
Platelet (x10^3^/μL)	133 (24 – 537)	227/348 (65.2)
C-reactive protein (mg/L)	64.2 (0.15 –>200)	300/308 (97.4)
Positive results of Scrub typhus antibody test	265 (75.9)	
Days of hospitalization	5 (2 – 208)	

Values are presented as number (%) or mean ± SD.

AST, aspartate aminotransferase; ALT, alanine aminotransferase; ALP, alkaline phosphatase;

PT(INR), prothrombine time-international normalized ratio.

### Hepatic dysfunction

Most patients with scrub typhus had hepatocellular-patterned dysfunction. The proportion of aminotransferases abnormalities (78.5% of AST and 63.0% of ALT >40 IU/L) was greater than the proportion of patients with abnormal ALP (27.2% >130 IU/L) and total bilirubin (16.1% >1.2 mg/dL) (Table-I).

### Abdominal CT

**Fig.1:**
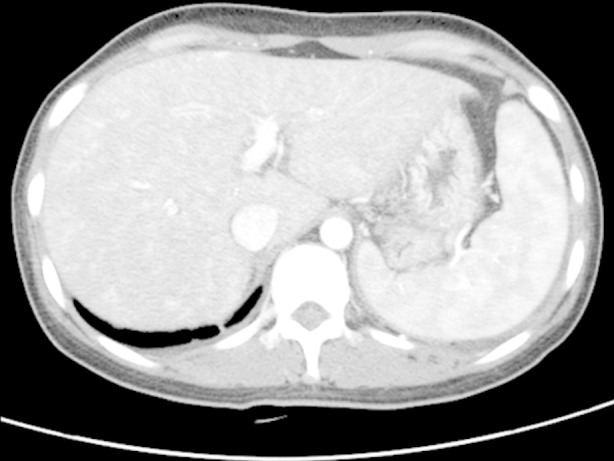
Scrub typhus in a 43-year-old woman. Arterial phase dynamic CT images show mild inhomogeneous enhancement of the hepatic parenchyma as well as splenomegaly.

**Fig.2:**
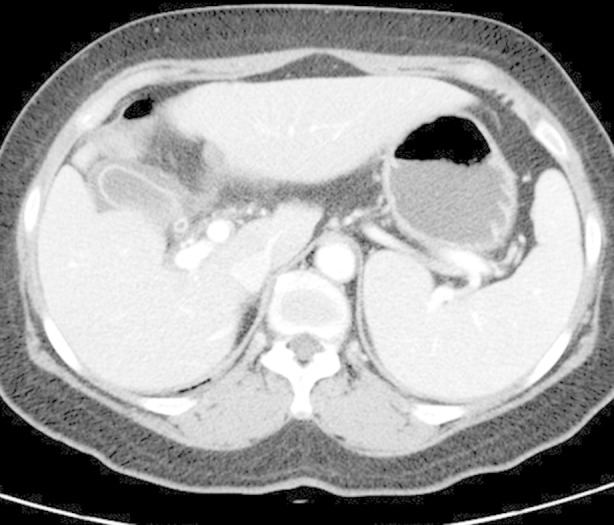
Scrub typhus in a 67-year-old woman. Serial contrast-enhanced abdominal CT images shows gallbladder wall thickening and splenomegaly.

94 of 349 patients underwent abdominal CT, mainly due to work-up for fever with hepatic dysfunction. Abdominal CT findings were enlarged lymph node (53.2%), inhomogeneous enhancement of liver (47.9%), splenomegaly (46.8%), ascites (28.7%), low attenuation of periportal areas (27.7%), gallbladder wall thickening (17.0%), and splenic infarct (6.4%). Ten patients (10.6%) had no abnormal findings of CT. There were no patients that presented all the aforementioned CT findings (Table-II, Fig. 1 and 2).

**Table-II:** Abdominal CT findings in 94 patients with scrub typhus.

Findings of CT	N (%)	Number of CT findings	N (%)
Enlarged lymph node	50 (53.2)	0	10 (10.6)
Inhomogeneous enhancement of liver	45 (47.9)	1	21 (22.3)
Splenomegaly	44 (46.8)	2	28 (29.8)
Ascites	27 (28.7)	3	14 (14.9)
Low attenuation of periportal areas	26 (27.7)	4	12 (12.8)
Gallbladder wall thickening	16 (17.0)	5	5 (5.3)
Splenic infarct	6 (6.4)	6	4 (4.3)
		7	0 (0.0)

Enlarged lymph node is defined as short-axis diameter of more than 10mm.

Splenomegaly is defined as maximal width of more than 11cm on the axial scan.

### Comparison of hepatic dysfunction according to the number of CT findings

Patients who underwent CT were classified into four groups according to the number of CT findings: ‘0’, 10 of 94 (10.6%); ‘1’, 21 of 94 (22.3%); ‘2’, 28 of 94 (29.8%); ‘3 or more’, 35 of 94 (37.2%). We compared the relationship between these groups and all parameters of Table-I. The aminotransferases increased according to the number of CT findings and were log transformed for the parametric statistics (AST, *P* = 0.028; log AST, *P*=0.011) (Table-III). Post-hoc analysis of the log AST revealed a significant difference in the mean difference between the ‘0’ and ‘3 or more’ groups (*P* = 0.041).

**Table-III:** Level of aminotranferase according to the number of abdominal CT findings (N = 94)

Number of CT findings	0	1	2	3 or more	P
N (%)	10 (10.6)	21 (22.3)	28 (29.8)	35 (37.2)	
AST (IU/L)	77.7±56.5	116.3±100.1	128.6±137.0	282.3±465.1	0.028
Log AST	1.8004±0.28941	1.9608±0.29214	1.9234±0.41122	2.1857±0.43884	0.011
ALT (IU/L)	66.6±54.4	89.2±81.4	97.4±128.9	221.2±443.0	0.103
Log ALT	1.6801±0.39648	1.8295±0.31212	1.7486±0.44636	2.0151±0.49694	0.051

Values are presented as mean ± SD.

P-values derived from the Kruskal-Wallis Test for AST and ALT.

P-values derived from the one-way ANOVA after transformed log for parametric statistics.

## DISCUSSION

Scrub typhus is endemic in Korea, China, Taiwan, Japan, Pakistan, India, Thailand, Malaysia and Australia, which form an area known as the “tsutsugamushi triangle”. It is a burden to the communities in Asia-Pacific rim.^12^-^15^ It also occur in the United States, Canada, and Europe, being imported by tourists returning from endemic regions.^16^

Previous studies have reported taht hepatic dysfunction in patients with scrub typhus was found to be a hepatocellular-patterned abnormality.^3^-^6^ This was ascertained by our study as well. Elevation of AST (78.5%) and ALT (63.0%) markedly exceeded those of ALP (27.2%) and total bilirubin (16.1%) (Table-I). The mechanism of this characteristic hepatocellular-patterned dysfunction is not clear, but is speculated pathologically. The infection of *Orientia tsutsugamushi* is characterized pathologically by focal or disseminated vasculitis and perivasculitis on the involved organs. Thus, scrub typhus infected in liver is speculated to cause mild focal inflammation due to intrahepatic sinusoidal endothelial vasculitis and to increase the levels of aminotransferases due to direct cytopathic liver damage.^4^,^17^,^18^

In 2007, Jeong et al. first described abdominal CT findings of scrub typhus with a small sample size (19 patients).^9^ In our study, abdominal CT images of 94 patients, more than in the previous study, were retrospectively reviewed by one radiologist. The proportions of CT findings were slightly different from the previous study. The most common findings were the enlarged lymph node (53.2%) in our study and splenomegaly (79%) in the previous study. However, inhomogeneous enhancement of liver, the secondary common finding in our study was 47.9%, virtually the same as the previous study (47%) (Table-II). These differences may be caused by the scale of studies and need further studies. In general, there was no newly detected CT finding. We also found that the radiologic findings of our cases were varied and not specific.

Furthermore, in endemic areas, it is not difficult to diagnose scrub typhus when an eschar is present^2^,^5^,^6^,^19^ or when the serologic test is positive.^13^,^20^-^22^ In our study, 245 of 345 patients (70.2%) presented with an eschar, and 265 of 345 patients (75.9%) exhibited positive serology were similar to those of previous studies. However, 94 patients who underwent the CT had either an eschar (50 of 94) or the positive result of serologic test (84 of 94). We know that serologic assay is the mainstay of diagnosis for scrub typhus. However, in patients who lack an eschar or present with negative serology, the differential diagnosis remains broad including a number of different diseases such as viral hepatitis or tuberculosis. The evaluation should consider the patient’s risk factors for liver disease as well as findings from the physical examination that may indicate a particular diagnosis. Through careful initial examination, we excluded other possible causes.

Additionally, the level of AST and log AST, transformed for the parametric statistics, was mostly elevated according to the number of CT findings (*P* = 0.028). Although both the ALT and log ALT were not statistically significant according to the number of CT findings (*P* = 0.103 and *P* = 0.051, respectively), these showed the tendency of increasing similar to the log AST in a rough way (Table-III). However, the clinical significance of this finding is unclear.

This study has some limitations. First, this is a single-center study of a relatively small number of patients with deranged aminotransferases. Second, the retrospective data collection is another limitation. So the generalizability of the radiologic manifestations of scrub typhus remain unclear. Further studies are needed to find out that which abdominal CT findings are suggestive of scrub typhus with hepatic dysfunction in conjunction with the patient’s history and clinical features and results of serologic testing.

## CONCLUSION

Our study showed that the most patients with scrub typhus had the hepatocellular -patterned dysfunction with a predominant elevation AST and ALT. Also, the abdominal CT features were enlarged lymph node, inhomogeneous enhancement of liver, splenomegaly, ascites, low attenuation of periportal areas, gallbladder wall thickening, and splenic infarct. We found that abdominal CT manifestations of scrub typhus with elevated aminotransferases were varied and not specific. However, knowledge of these findings may evoke the recognition of scrub typhus by clinicians in endemic areas.

## References

[1] Chi WC, Huang JJ, Sung JM, Lan RR, Ko WC, Chen FF (1997). Scrub typhus associated with multiorgan failure: a case report. Scand J Infect Dis.

[2] Park JI, Han SH, Cho SC, Jo YH, Hong SM, Lee HH (2003). Outbreak of hepatitis by Orientia tsutsugamushi in the early years of the new millenium. Taehan Kan Hakhoe Chi.

[3] Hu ML, Liu JW, Wu KL, Lu SN, Chiou SS, Kuo CH (2005). Short report: Abnormal liver function in scrub typhus. Am J Trop Med Hyg.

[4] Kanno A, Yamada M, Murakami K, Torinuki W (1996). Liver involvement in Tsutsugamushi disease. Tohoku J Exp Med.

[5] Vivekanandan M, Mani A, Priya YS, Singh AP, Jayakumar S, Purty S (2010). Outbreak of scrub typhus in Pondicherry. J Assoc Physicians India.

[6] Yang CH, Hsu GJ, Peng MY, Young TG (1995). Hepatic dysfunction in scrub typhus. J Formos Med Assoc.

[7] Koh GC, Maude RJ, Paris DH, Newton PN, Blacksell SD (2010). Diagnosis of scrub typhus. Am J Trop Med Hyg.

[8] Nachega JB, Bottieau E, Zech F, Van Gompel A (2007). Travel-acquired scrub typhus: emphasis on the differential diagnosis, treatment, and prevention strategies. J Travel Med.

[9] Jeong YJ, Kim S, Wook YD, Lee JW, Kim KI, Lee SH (2007). Scrub typhus: clinical, pathologic, and imaging findings. Radiographics.

[10] Lee KD, Moon C, Oh WS, Sohn KM, Kim BN (2014). Diagnosis of scrub typhus: introduction of the immunochromatographic test in Korea. Korean J Intern Med.

[11] Bezerra AS, D’Ippolito G, Faintuch S, Szejnfeld J, Ahmed M (2005). Determination of splenomegaly by CT: is there a place for a single measurement?. Am J Roentgenol.

[12] Kelly DJ, Fuerst PA, Ching WM, Richards AL (2009). Scrub typhus: the geographic distribution of phenotypic and genotypic variants of Orientia tsutsugamushi. Clin Infect Dis.

[13] Olson JG, Bourgeois AL (1977). Rickettsia tsutsugamushi infection and scrub typhus incidence among Chinese military personnel in the Pescadores Islands. Am J Epidemiol.

[14] Phongmany S, Rolain JM, Phetsouvanh R, Blacksell SD, Soukkhaseum V, Rasachack B (2006). Rickettsial infections and fever, Vientiane, Laos. Emerg Infect Dis.

[15] Noh M, Lee Y, Chu C, Gwack J, Youn SK, Huh S (2013). Are there spatial and temporal correlations in the incidence distribution of scrub typhus in Korea?. Osong Public Health Res Perspect.

[16] Jensenius M, Fournier PE, Raoult D (2004). Rickettsioses and the international traveler. Clin Infect Dis.

[17] Watanabe H, Saito T, Misawa K, Suzuki A, Sanjo M, Okumoto K (2005). Direct cytopathic liver injury and acute respiratory distress syndrome associated with gilliam-type tsutsugamushi disease. J Gastroenterol Hepatol.

[18] Chung JH, Lim SC, Yun NR, Shin SH, Kim CM, Kim DM (2012). Scrub typhus hepatitis confirmed by immunohistochemical staining. World J Gastroenterol.

[19] Watt G, Parola P (2003). Scrub typhus and tropical rickettsioses. Curr Opin Infect Dis.

[20] Blacksell SD, Paris DH, Chierakul W, Wuthiekanun V, Teeratakul A, Kantipong P (2012). Prospective evaluation of commercial antibody-based rapid tests in combination with a loop-mediated isothermal amplification PCR assay for detection of Orientia tsutsugamushi during the acute phase of scrub typhus infection. Clin Vaccine Immunol.

[21] Silpasakorn S, Srisamut N, Ekpo P, Zhang Z, Chao CC, Ching WM (2012). Development of new, broadly reactive, rapid IgG and IgM lateral flow assays for diagnosis of scrub typhus. Am J Trop Med Hyg.

[22] Silpasakorn S, Waywa D, Hoontrakul S, Suttinont C, Losuwanaluk K, Suputtamongkol Y (2012). Performance of SD Bioline Tsutsugamushi assays for the diagnosis of scrub typhus in Thailand. J Med Assoc Thai.

